# Clinimetric Evaluation of the Experienced Communication in Dementia Questionnaire

**DOI:** 10.1093/geront/gnab187

**Published:** 2021-12-28

**Authors:** Maria W L J Olthof-Nefkens, Els W C Derksen, Britt Lambregts, Bert J M de Swart, Maria W G Nijhuis-van der Sanden, Johanna G Kalf

**Affiliations:** Zorggroep Maas & Waal, Beneden-Leeuwen, The Netherlands; Department of Rehabilitation, Donders Institute for Brain, Cognition and Behaviour, Radboud University Medical Center, Nijmegen, The Netherlands; Department of Primary and Community Care, Radboud Institute for Health Sciences, Radboud University Medical Center, Nijmegen, The Netherlands; Radboudumc Alzheimer Center, Radboud University Medical Center, Nijmegen, The Netherlands; Donders Institute for Brain, Cognition and Behaviour, Radboud University, Nijmegen, The Netherlands; Department of Psychiatry, Radboud University Medical Center, Nijmegen, The Netherlands; Department of Rehabilitation, Donders Institute for Brain, Cognition and Behaviour, Radboud University Medical Center, Nijmegen, The Netherlands; Radboudumc Alzheimer Center, Radboud University Medical Center, Nijmegen, The Netherlands; Radboud Institute for Health Sciences, Scientific Center for Quality of Healthcare (IQ Healthcare), Radboud University Medical Center, Nijmegen, The Netherlands; Department of Rehabilitation, Donders Institute for Brain, Cognition and Behaviour, Radboud University Medical Center, Nijmegen, The Netherlands; Radboudumc Alzheimer Center, Radboud University Medical Center, Nijmegen, The Netherlands

**Keywords:** Alzheimer’s disease, Caregiver, Cognitive communication disorders, Patient-reported outcome measures, Speech and language therapy

## Abstract

**Background and Objectives:**

Tools to measure self-perceived communication between persons with early-stage dementia and their caregivers are lacking. Therefore, we developed a questionnaire for Experienced Communication in Dementia (ECD) with a patient version (ECD-P) and a caregiver version (ECD-C), which contains items on (a) caregiver competence, (b) social communication, (c) communication difficulties, and (d) experienced emotions. This article describes the feasibility and clinimetric evaluation of this instrument.

**Research Design and Methods:**

A prospective observational cohort study was conducted with 57 dyads (community-dwelling person with dementia and primary caregiver). ECD-P, ECD-C, and measures on quality of life, caregiver burden, cognitive functioning, physical functioning, and functional independence were administered. After 2 weeks, the dyads filled out the ECD again. Feasibility (completion time and missing values per item), internal consistency (Cronbach’s α), test–retest reliability (intraclass correlation coefficients [ICCs]), and construct validity (hypotheses testing with Spearman’s *r*) were evaluated.

**Results:**

Mean completion time was 10 min per questionnaire. ICCs for test–retest reliability ranged from 0.67 to 0.78, except for ECD-P2 (ICC = 0.31). Internal consistency ranged from α = 0.75 to 0.82 for ECD-P1 and all parts of ECD-C, except for ECD-P2 (α = 0.66). Correlation coefficients for convergent validity ranged from *r* = 0.31 to 0.69 and correlation coefficients for divergent validity were *r* < 0.20 and statistically insignificant.

**Discussion and Implications:**

Pending future research, the ECD, except part ECD-P2, seems to be a promising tool to measure experienced communication between persons with early-stage dementia and their caregivers.

## Background and Objectives

Cognitive communication disorders (CCDs) are very common in people with dementia (Bayles & Tomoeda, 2014), and although not always acknowledged, they are often present from an early stage (Gauthier, 2001). The various manifestations of CCD pose daily challenges to all persons involved. They can cause increased misunderstanding and frustration, gradually leading to restricted social participation of the person with dementia (Schoenmakers et al., 2010), and CCDs have also been found to contribute to caregiver burden (Stiadle et al., 2014). Therefore, there is a growing interest in the development of communication interventions that may prevent or relieve these troublesome effects of CCD (Barnes & Markham, 2018; Williams et al., 2018) and subsequently in communication-related measurements that are able to properly evaluate these interventions (Eadie et al., 2006; Haberstroh et al., 2013; Williams et al., 2017; Williams & Parker, 2012).

Currently, a new short-term logopedic intervention (Olthof-Nefkens et al., 2018) for people with dementia and their caregivers is being evaluated at the Radboudumc in Nijmegen, The Netherlands. The goal of this intervention is to optimize the communication between people with dementia and their caregivers, thus having a positive impact on how they experience their communication with each other and with the people in their social environment.

However, despite intensive searching, we found no instrument that is supposed to measure how people experience their own communication. We found only a few dementia-specific instruments, and although each of them measures a relevant construct, such as either language performance (Bayles & Tomoeda, 1993; Ferris et al., 2009; Rousseaux et al., 2010), functional communication (Bayles & Tomoeda, 1994; Volicer & Manzar, 2018), or communication ability (Strøm et al., 2016), they do not measure people’s personal experiences. Also, they are usually filled out by informal caregivers (proxy measures) or health care professionals, thereby neglecting valuable input from the persons with dementia themselves.

Therefore, we constructed a questionnaire to evaluate the impact of the new logopedic intervention: the “Experienced Communication in Dementia questionnaire” (ECD), with a version for the person with dementia and a version for the caregiver. The ECD was developed in close collaboration with both people with dementia and their caregivers as well as with experts in the field of dementia care. We interviewed five person with dementia—caregiver dyads who had recently received the logopedic intervention. We asked them questions about the communication difficulties they encountered (e.g., barriers and facilitators, experienced emotions, needs) and to tell us about the impact of the intervention on their lives (e.g., changes that occurred on behavior and emotions, experiences with given advice). We performed reflexive thematic analysis on the transcripts of the interviews and generated items for the questionnaires. While formulating the items, we tried to stay close to the words that were used by the participants. Then, we selected items and response scales in collaboration with the same dyads. The final version of the questionnaire was established after pilot testing with seven other dyads and discussion with five experts in the field of dementia care. More details on the development and face validity of the questionnaire are described elsewhere (Olthof-Nefkens et al., 2021). This previous study showed that how people with dementia experience their communication is defined by a combination of four factors: the communicative competence of the conversation partner, their communication behaviors in social settings, the communication difficulties they experience in daily life, and the emotions they have during conversations (nervousness, frustration, sadness, anger, and anxiety; Olthof-Nefkens et al., 2021). These themes correspond with the domains that are targeted in the intervention and are therefore embedded in the ECD.

The aim of this article is to report about the feasibility of the ECD and its clinimetric properties, being internal consistency, test–retest reliability, and construct validity.

## Research Design and Methods

### Design

A prognostic observational cohort study was conducted, using the *Consensus-based standards for the selection of health measurement instruments* taxonomy and definitions (Mokkink et al., 2010) to determine feasibility, internal consistency, test–retest reliability, and construct validity of the “Experienced Communication in Dementia” questionnaire.

### Participants

Participants were recruited from the Geriatrics Department of the Radboudumc in Nijmegen (The Netherlands), visiting between September 2015 and January 2016. Inclusion criteria were (a) diagnosed with mild to moderate dementia (Clinical Dementia Rating score between 0.5 and 2; Rikkert et al., 2011) by a geriatrician, (b) home-living with a primary caregiver, and (c) being able to read and understand Dutch. Exclusion criteria were uncorrected visual or hearing impairment and medical or psychiatric comorbidity (e.g., stroke, major depression) that could limit the ability to participate in the study.

### Procedure

Participants were selected from dyads (person with dementia and primary caregiver) that were already invited to the outpatient clinic of the Geriatrics Department of the Radboudumc for a routine follow-up appointment. One week before this consultation, a letter with information about this study was sent to these dyads, including the measurement procedure and request for participation. The dyads were asked to notify the geriatrician or physician assistant whether or not they agreed to participate and at the end of their consultation, the geriatrician or physician assistant asked the dyads whether they still wanted to participate in this study. If so, they were introduced to researcher B. Lambregts.

After signing informed consent forms, the person with dementia and the caregiver individually completed the ECD and the Dementia Quality of life Instrument (DQI; Schölzel-Dorenbos et al., 2012). The caregivers also completed the Zarit Burden Interview Short Form (ZBI-12; Bédard et al., 2001). The task of the researcher was to make sure that the person with dementia and caregiver did not interact during the completion of the questionnaires and to neutrally explain the questions and answering options to the person with dementia if needed, without influencing the responses.

To evaluate test–retest reliability, the participants were asked to complete the ECD for a second time after 2 weeks. These questionnaires were sent and returned by postal mail. Participants were asked to complete the questionnaires independently; without interaction with each other, except when help was necessary for understanding all questions. Although we could not control the situation at people’s homes, the written instructions were as similar as possible to the ones during the first measurement. We chose a time frame of 2 weeks based on the assumption that this was long enough to prevent recall bias and short enough to ensure that dementia had not worsened dramatically in between (Terwee et al., 2007).

### Ethics

This study was approved by the regional medical ethics committee (file number 2014-1225), and all people with dementia and caregivers signed an informed consent form.

### Experienced Communication in Dementia Questionnaire (ECD-P and ECD-C)

The patient version of the ECD (ECD-P) consists of two parts with a total of 24 items (Table 1). The first part (22 items) contains items in the four themes that define the construct of “experienced communication” (caregiver competence, social communication, communication difficulties in daily life, and experienced emotions during conversations) and is considered to be “the body” of the instrument. Response options are 4-point Likert scales, either for agreement (fully disagree–partially disagree–partially agree–fully agree) or for frequency (during every conversation–every day–every week–(almost) never). Possible scores range from 0 to 66, with lower scores reflecting a more positive experienced communication.

**Table 1.** Experienced Communication in Dementia Questionnaire—Patient Version

Themes	Items	Response options	Scores
Part 1			
Caregiver competence	1. My caregiver makes an effort to understand me	Strongly disagree–disagree–agree–strongly agree	3–2–1–0
	2. My caregiver usually talks at a pleasant pace (not too fast and not too slow)	Strongly disagree–disagree–agree–strongly agree	3–2–1–0
	3. My caregiver makes eye contact when we talk to each other	Strongly disagree–disagree–agree–strongly agree	3–2–1–0
	4. I feel safe in conversations where my caregiver is present	Strongly disagree–disagree–agree–strongly agree	3–2–1–0
	5. My caregiver and I talk less and less to each other	Strongly disagree–disagree–agree–strongly agree	0–1–2–3
Social communication	6. I have become more quiet than I used to be	Strongly disagree–disagree–agree–strongly agree	0–1–2–3
	7. I tend to withdraw from conversations	Strongly disagree–disagree–agree–strongly agree	0–1–2–3
	8. I try to avoid events where there are many people present	Strongly disagree–disagree–agree–strongly agree	0–1–2–3
	9. I like to be helped when I experience communication breakdown	Strongly disagree–disagree–agree–strongly agree	3–2–1–0
	10. I tell people when I get stuck in a conversation	Strongly disagree–disagree–agree–strongly agree	3–2–1–0
	11. I tell people about my illness	Strongly disagree–disagree–agree–strongly agree	3–2–1–0
	12. People adjust to the way I communicate	Strongly disagree–disagree–agree–strongly agree	3–2–1–0
	13. I am satisfied with my current social contacts	Strongly disagree–disagree–agree–strongly agree	3–2–1–0
	14. Friends and acquaintances come to visit as often as they did in the past	Strongly disagree–disagree–agree–strongly agree	3–2–1–0
Communication difficulties in daily life	15. I cannot find the right words	During every conversation–every day–every week–(almost) never	3–2–1–0
	16. I am not able to participate because the conversation goes too fast	During every conversation–every day–every week–(almost) never	3–2–1–0
	17. There are misunderstandings between me and my caregiver	During every conversation–every day–every week–(almost) never	3–2–1–0
Experienced emotions of persons living with dementia	18. I feel nervous during a conversation	During every conversation–every day–every week–(almost) never	3–2–1–0
	19. I feel frustrated during a conversation	During every conversation–every day–every week–(almost) never	3–2–1–0
	20. I feel sad during a conversation	During every conversation–every day–every week–(almost) never	3–2–1–0
	21. I feel angry during a conversation	During every conversation–every day–every week–(almost) never	3–2–1–0
	22. I feel anxious during a conversation	During every conversation–every day–every week–(almost) never	3–2–1–0
Part 2			
Assessment of conversation quality	23. In general, I would grade the conversations between me and my partner with an:	(Poor) 1–2–3–4–5–6–7–8–9–10 (Excellent)	
	24. In general, I would grade the conversations between me and the people in our immediate surroundings (children, friends, neighbors, etc.) with an:	(Poor) 1–2–3–4–5–6–7–8–9–10 (Excellent)	

Part 2 contains two items for an overall judgment of the conversation quality (a) between the person with dementia and the caregiver and (b) between the person with dementia and closest family members and friends. The response is scored on a 10-point scale from 1 (*poor*) to 10 (*excellent*). Sum scores range from 2 to 20, with higher scores indicating a more positive experienced communication.

The caregiver version of the ECD (ECD-C) is similar (Table 2), but with all items formulated to represent the experiences of the person with dementia from the perspective of the caregiver (e.g., “I feel nervous during a conversation” in ECD-P is formulated as “My partner feels nervous during a conversation” in ECD-C). Scores range identical to Parts 1 and 2 of the ECD-P. This version has an additional third part of five items about the caregiver’s experienced emotions regarding the communication problems. These items have the same 4-point response scales as Part 1, for either agreement or frequency. Possible scores range from 0 to 15, with lower scores reflecting a more positive experienced communication. The ECD-C has a total of 29 items. The items were translated into English by the first author for publishing purposes only.

**Table 2.** Experienced Communication in Dementia Questionnaire—Caregiver Version

Themes	Items	Response options	Scores
Part 1			
Caregiver competence	1. I make an effort to understand my partner	Strongly disagree–disagree–agree–strongly agree	3–2–1–0
	2. I usually talk at a pleasant pace (not too fast and not too slow)	Strongly disagree–disagree–agree–strongly agree	3–2–1–0
	3. I make eye contact with my partner when we talk to each other	Strongly disagree–disagree–agree–strongly agree	3–2–1–0
	4. My partner feels safe in conversations where I am present	Strongly disagree–disagree–agree–strongly agree	3–2–1–0
	5. My partner and I talk less and less to each other	Strongly disagree–disagree–agree–strongly agree	0–1–2–3
Social communication	6. My partner has become more quiet than he/she used to be	Strongly disagree–disagree–agree–strongly agree	0–1–2–3
	7. My partner tends to withdraw from conversations	Strongly disagree–disagree–agree–strongly agree	0–1–2–3
	8. My partner tries to avoid events where there are many people present	Strongly disagree–disagree–agree–strongly agree	0–1–2–3
	9. My partner likes to be helped when he/she experiences communication breakdown	Strongly disagree–disagree–agree–strongly agree	3–2–1–0
	10. My partner tells people when he/she gets stuck in a conversation	Strongly disagree–disagree–agree–strongly agree	3–2–1–0
	11. My partner tells people about his/her illness	Strongly disagree–disagree–agree–strongly agree	3–2–1–0
	12. People adjust to the way my partner communicates	Strongly disagree–disagree–agree–strongly agree	3–2–1–0
	13. My partner is satisfied with his/her current social contacts	Strongly disagree–disagree–agree–strongly agree	3–2–1–0
	14. Friends and acquaintances come to visit as often as they did in the past	Strongly disagree–disagree–agree–strongly agree	3–2–1–0
Communication difficulties in daily life	15. My partner cannot find the right words	During every conversation–every day–every week–(almost) never	3–2–1–0
	16. My partner is not able to participate because the conversation goes too fast	During every conversation–every day–every week–(almost) never	3–2–1–0
	17. There are misunderstandings between me and my partner	During every conversation–every day–every week–(almost) never	3–2–1–0
Experienced emotions of persons living with dementia	18. My partner feels nervous during a conversation	During every conversation–every day–every week–(almost) never	3–2–1–0
	19. My partner feels frustrated during a conversation	During every conversation–every day–every week–(almost) never	3–2–1–0
	20. My partner feels sad during a conversation	During every conversation–every day–every week–(almost) never	3–2–1–0
	21. My partner feels angry during a conversation	During every conversation–every day–every week–(almost) never	3–2–1–0
	22. My partner feels anxious during a conversation	During every conversation–every day–every week–(almost) never	3–2–1–0
Part 2			
Assessment of conversation quality	23. In general, I would grade the conversations between me and my partner with an:	(Poor) 1–2–3–4–5–6–7–8–9–10 (Excellent)	
	24. In general, I would grade the conversations between my partner and the people in our immediate surroundings (children, friends, neighbors, etc.) with an:	(Poor) 1–2–3–4–5–6–7–8–9–10 (Excellent)	
Part 3			
Experienced emotions of caregiver	25. I find it tiring to interact with my partner	Strongly disagree–disagree–agree–strongly agree	0–1–2–3
	26. It burdens me that communication is becoming increasingly difficult	Strongly disagree–disagree–agree–strongly agree	0–1–2–3
	27. I feel angry during a conversation	During every conversation–every day–every week–(almost) never	3–2–1–0
	28. I feel sad during a conversation	During every conversation–every day–every week–(almost) never	3–2–1–0
	29. I feel frustrated during a conversation	During every conversation–every day–every week–(almost) never	3–2–1–0

The content of Tables 1 and 2 (hyperlink: https://journals.sagepub.com/doi/pdf/10.1177/00469580211028181) by M.W.L.J. Olthof-Nefkens et al. is licensed under CC BY 4.0 (hyperlink: https://creativecommons.org/licenses/by/4.0/).

Formatted versions that were used by the participants (without scores) are available as supplementary materials (Supplementary Sections I and II).

### Clinimetric Evaluation

#### Feasibility

The feasibility of the ECD was evaluated in terms of the time needed to complete the questionnaires. Also, the percentage of missing values per item was registered, first to get an indication of items that still may be difficult to score, second to be able to calculate if and how to complete missing items for calculations of total scores.

#### Internal consistency

Internal consistency was evaluated for all parts of the ECD to determine the homogeneity of the constructs of experienced communication (ECD-P1 and ECD-C1), experienced quality of conversations (ECD-P2 and ECD-C2), and caregiver’s experienced emotions (ECD-C3).

#### Test–retest reliability

Test–retest reliability refers to the reproducibility of the questionnaire and was based on the measurement of the same person on two occasions in the same health status with the same instrument. To evaluate this feature, the participants were asked to complete the ECD for a second time after 2 weeks.

#### Construct validity

Following the description of the content validity as reported in our previously published paper on this instrument (Olthof-Nefkens et al., 2021) and assuming acceptable internal consistency, we aimed to investigate construct validity by comparing the ECD scores with measures that we anticipated to correlate with the construct of “experienced communication.” Measures had to be available and validated in Dutch and their use without additional burden for the participants. We were unable to find another Dutch instrument that measures a construct close to “experienced communication”; therefore, we decided to take the Dutch versions of the DQI and the ZBI-12 as the best possible convergent measures that met our requirements. The DQI is a dementia-specific health-related quality of life index measure (Schölzel-Dorenbos et al., 2012) that consists of six items; a higher score indicates a higher health-related quality of life. The DQI was completed by both the person (DQI-P) with dementia and the caregiver (DQI-C). The ZBI-12 is a 12-item questionnaire about caregiver burden (Bédard et al., 2001) with higher scores suggesting higher caregiver burden. The ZBI-12 was completed by the caregivers only.

We chose the Mini-Mental State Examination (MMSE), Barthel Activities of Daily Living (ADL) Index, and Lawton Instrumental Activities of Daily Living (IADL) as divergent measures for the ECD. The MMSE is widely used to score and interpret older people’s cognitive function (Vertesi et al., 2001). The MMSE consists of 20 items and higher scores indicate better cognitive functioning. The Barthel ADL Index is a generally used instrument to measure the level of functional independence on everyday tasks (Mahony & Barthel, 1965; Wade & Collin, 1988). The ADL consists of 10 items and a higher score is a reflection of greater ability to function independently. The Lawton IADL scale was used for measuring physical functioning (Graf, 2008; Lawton & Brody, 1969). The IADL consists of eight items and higher scores indicate better independent living skills. These three measures were administered by the geriatrician or physician assistant as part of the standard clinical consultation. All measures were collected on the same day.

### Statistical Analyses

We used IBM SPSS Statistics version 25 for all calculations and accepted *p* values of <.05 (two-tailed) to be statistically significant.

Because all parts of the ECD have their own scoring (4-point items vs. 10-point items), a total score of all parts is not possible. Therefore, sum scores of the parts were used in all calculations. Because sum scores can only be calculated when all items have a response, a Missing Values Analysis was conducted in SPSS to test the hypothesis that the missing data were missing completely at random (Little’s MCAR test; Little, 1988). If *p* values were larger than .05, missing values were indeed missing completely at random, and no subsequent analyses were needed. The “expectation-maximization procedure” in SPSS was then used to calculate participants’ mean score for all completed items and replace missing values in the data set with these estimated values.

For evaluation of internal consistency, Cronbach’s α was calculated for each part of the ECD, accepting values between 0.70 and 0.95 (Terwee et al., 2007). We considered floor and ceiling effects to be acceptable when less than 15% of the persons scored either the lowest or highest possible score (Terwee et al., 2007).

To evaluate test–retest reliability, intraclass correlation coefficients (ICCs) estimates and their 95% confidence intervals were calculated based on a single measurement, absolute-agreement, and two-way mixed-effects model (Koo & Li, 2016). Reliability was considered poor with an ICC lower than 0.50, moderate with an ICC between 0.50 and 0.75, and good with an ICC higher than 0.75 (Koo & Li, 2016).

Construct validity was investigated by associating the scores on each part of the ECD-P and ECD-C with each other, as well as associating the ECD scores with the convergent and divergent measures. Correlations were calculated by using Spearman’s correlation coefficient (*r*). We a priori judged coefficients less than 0.30 as weak, 0.30–0.70 as substantial, and larger than 0.70 as strong (Aday & Cornelius, 2006). Hypotheses about direction and magnitude of correlations between measurements were formulated a priori (Table 5). We anticipated that the two parts of the ECD-P and the three parts of the ECD-C would correlate at least substantially with each other, because they measure the same construct, but from different perspectives (self-report vs. proxy report). We did not expect a high correlation because dementia care research on quality of life questionnaires has shown that people with dementia tend to give higher scores to their quality of life than their caregivers do (Logsdon et al., 1999; Römhild et al., 2018). This phenomenon might also occur on ECD scores. We expected the correlations between ECD-P Parts 1 and 2 with the DQI-P to be substantial, because communication problems are assumed to have an influence on the experienced quality of life of persons with dementia (Banerjee et al., 2010; Yorkston et al., 2010) while the ability to interact with the environment is described as a part of the conceptual framework of quality of life (Brod et al., 1999). The correlations between all three parts of the ECD-C with the DQI-C were hypothesized to be lower, but still substantial, because we expected communication problems of the persons with dementia to have a moderate impact on the overall quality of life of the caregivers (Banerjee et al., 2010; Stiadle et al., 2014). We also expected substantial correlations between all three parts of ECD-C with the scores on the ZBI-12, because CCDs have been found to contribute considerably to caregiver burden (Savundranayagam et al., 2005; Stiadle et al., 2014). Scores on MMSE, ADL, and IADL were expected to have a weak correlation with ECD-P1 and ECD-C1 scores. Although communication and cognition are highly interdependent constructs and language performance decreases when disease severity increases (Bayles & Tomoeda, 2014), the ECD does not measure communication skills itself, but the perceived impact of communication difficulties. We assume that the way in which people experience their communication is more related to contextual and personal factors, like the quality of relationships, than to cognitive or physical functioning of the person with dementia (Hernandez et al., 2019).

**Table 5.** Construct Validity of the Two Parts of the Experienced Communication in Dementia Questionnaire (ECD-P1 and ECD-P2) Patient Version and the Three Parts of the Caregiver Version (ECD-C1, ECD-C2, ECD-C3) Based on 57 Dyads

Measures		Correlation hypothesis (direction/magnitude)	Result (Spearman’s *r*)	p	Confirmed (yes/no)
ECD-P1	ECD-P2	Negative/substantial	−0.41	.00	Yes
	ECD-C1	Positive/substantial	0.55	.00	Yes
	ECD-C2	Negative/substantial	−0.31	.02	Yes
	ECD-C3	Positive/substantial	0.32	.00	Yes
ECD-P2	ECD-C1	Negative/substantial	−0.32	.02	Yes
	ECD-C2	Positive/substantial	0.23	.08	No
	ECD-C3	Negative/substantial	−0.20	.14	No
ECD-C1	ECD-C2	Negative/substantial	−0.69	.00	Yes
	ECD-C3	Positive/substantial	0.50	.00	Yes
ECD-C2	ECD-C3	Negative/substantial	−0.54	.00	Yes
DQI-P	ECD-P1	Negative/substantial	−0.53	.00	Yes
	ECD-P2	Positive/substantial	−0.03	.85	No
DQI-C	ECD-C1	Negative/substantial	−0.44	.00	Yes
	ECD-C2	Positive/substantial	0.40	.00	Yes
	ECD-C3	Negative/substantial	−0.47	.00	Yes
ZBI-12	ECD-C1	Positive/substantial	0.36	.01	Yes
	ECD-C2	Negative/substantial	−0.45	.00	Yes
	ECD-C3	Positive/substantial	0.50	.00	Yes
MMSE	ECD-P1	Negative/weak	−0.01	.97	No
	ECD-C1	Negative/weak	−0.13	.92	No
ADL	ECD-P1	Negative/weak	0.09	.48	No
	ECD-C1	Negative/weak	−0.11	.42	No
IADL	ECD-P1	Negative/weak	−0.20	.13	No
	ECD-C1	Negative/weak	0.17	.20	No

*Notes:* ECD-P = Experienced Communication in Dementia questionnaire, patient version, two parts; ECD-C = Experienced Communication in Dementia questionnaire, caregiver version, three parts; DQI-P = Dementia Quality of life Instrument by the person with dementia; DQI-C = Dementia Quality of life Instrument by the caregiver; ZBI-12 = Zarit Burden Interview Short Form; MMSE = Mini-Mental State Examination; ADL = Barthel Activities of Daily Living Index; IADL = Lawton Instrumental Activities of Daily Living. Spearman’s *r*: <0.30 weak, 0.30–0.70 substantial, >0.70 strong.

## Results

### Participants

A total of 89 dyads were asked to participate, and 57 dyads (64%) agreed. Characteristics of the people with dementia are given in Table 3. There were slightly more men (58%) than women (42%). All caregivers were either partners, relatives, or close friends, with more women (68%) than men (32%).

**Table 3.** Participant Characteristics (*N* = 114)

Variable	N	%	Mean (min–max) ± *SD*
Sex of persons living with dementia			
Men	33	58	
Women	24	42	
Age of persons living with dementia (years)			76 (57–91) ± 7.3
Sex of caregivers			
Men	18	32	
Women	39	68	
Age of caregivers (years)			65 (41–86) ± 11.4
Education of persons living with dementia			
Primary school	6	10.3	
Prevocational secondary education	19	32.8	
Senior general secondary education	4	6.9	
Secondary vocational education	14	24.1	
Higher professional education	9	15.5	
University education	5	8.6	
Diagnosis			
Alzheimer’s disease	50	87.7	
Frontotemporal dementia	1	1.8	
Lewy body disease	1	1.8	
Primary progressive aphasia	1	1.8	
Mixed	4	7.0	
Clinical Dementia Rating scale			
0.5	2	3.4	
1.0	51	87.9	
2.0	5	8.6	
Disease duration (years)			2.4 (1–6) ± 1.5
DQI-P (range 0–1)			0.81 (0.01–0.99) ± 0.17
DQI-C (range 0–1)			0.97 (0.67–1.00) ± 0.08
ZBI-12 (range 0–48)			12.4 (0–36) ± 7.3
MMSE (range 0–30)			21.8 (7–29) ± 4.4
ADL (range 0–20)			19.7 (12–20) ± 1.2
IADL (range 0–8)			4.1 (0–8) ± 2.0

*Note:* DQI-P = Dementia Quality of life Instrument by the patient; DQI-C = Dementia Quality of life Instrument by the caregiver; ZBI-12 = Zarit Burden Interview Short Form; MMSE = Mini-Mental State Examination; ADL = Barthel Activities of Daily Living Index; IADL = Lawton instrumental activities of daily living.

### Feasibility

On average, persons with dementia were able to complete the questionnaire within 11 min (range 5–14 minutes). Caregivers needed an average time of 9 min (range 6–13 min).

For the first measurement, our data set contained 18 missing values (1.4%) on all items of ECD-P (*n* = 57) and 21 missing values (1.3%) on all ECD-C items (*n* = 57). For the second measurement, 31 values (2.8%) were omitted in the returned ECD-P (*n* = 45) and 44 (3%) missing values for ECD-C (*n* = 49).

Missing values per item ranged from 0% to 5.3%, with one outlier for Item 12, that was omitted by 17.5% of persons with dementia and 8.8% of caregivers. All *p* values for Little’s MCAR were >.05, ranging from 0.29 to 0.97, making it acceptable to execute the expectation-maximization procedure as planned and complete the data set with estimated values.

### Internal Consistency

ECD characteristics are given in Table 4. Internal consistency was good for Part 1 of the ECD-P and all parts of the ECD-C and moderate for Part 2 of the ECD-P.

**Table 4.** Scale Characteristics and Reproducibility

ECD-part (number of items)	Range	Mean (*SD*) first measurement	Internal consistency (Cronbach’s α)	Test–retest measurement	Test–retest (ICC and 95% CI)	Floor/ceiling effects (%)
ECD-P1 (22)	0–66	17.1 (6.3)	0.76	45	0.67 (0.48–0.80)	0.0/0.0
ECD-P2 (2)	2–20	15.7 (2.2)	0.66	44	0.31 (0.02–0.55)	0.0/1.8
ECD-C1 (22)	0–66	22.5 (7.6)	0.78	49	0.76 (0.61–0.85)	0.0/0.0
ECD-C2 (2)	2–20	13.6 (2.6)	0.82	45	0.75 (0.59–0.86)	0.0/1.8
ECD-C3 (5)	0–15	4.5 (2.6)	0.75	49	0.78 (0.64–0.86)	5.5/0.0

*Note:* ECD-P = Experienced Communication in Dementia questionnaire, patient version, two parts; ECD-C = Experienced Communication in Dementia questionnaire, caregiver version, three parts; ICC = intraclass correlation coefficient.

### Test–Retest Reliability

Of the 57 questionnaires that were sent for the second measurement, 45 ECD-P (79%) and 49 ECD-C (86%) were returned. Test–retest analysis revealed a good reliability for all three parts of the ECD-C and a moderate reliability for ECD-P Part 1, but reproducibility of ECD-P Part 2 turned out to be poor. No floor or ceiling effects were found (Table 4).

### Construct Validity

Table 5 displays the correlations between ECD-P (Parts 1 and 2), ECD-C (Parts 1, 2, and 3), and the other measurements. Regarding the instrument itself, ECD-P1 and ECD-P2, ECD-P1 and all parts of ECD-C, and ECD-P2 and ECD-C1 all correlate substantially. Against our expectations, no significant correlations were found between ECD-P2 and ECD-C2 or ECD-C3. ECD-C1 and ECD-C2 correlate strongly, while ECD-C3 correlates substantially with ECD-C1 and ECD-C2. Comparison of the ECD with the other measures revealed a substantial correlation between ECD-P1 and DQI-P, but no correlation between ECD-P2 and DQI-P. All three parts of ECD-C correlate substantially with both DQI-C and ZBI-12. We found no statistically significant correlations between ECD-P1 and ECD-C1 with MMSE, ADL, and IADL.

## Discussion and Implications

This clinimetric study shows that the Experienced Communication in Dementia questionnaire for persons with dementia (ECD-P) Part 1 and all parts of the Experienced Communication in Dementia questionnaire for caregivers (ECD-C) seem to be feasible and reliable for use in people with early-stage dementia and their caregivers. The second part of the ECD-P, however, lacks sufficient test–retest reliability and construct validity and is therefore not recommended for further use.

The ECD proved to take about 10 min to complete. During its development, the ECD was constructed in close collaboration with people with dementia and their caregivers, and their own words were used to formulate the items (Olthof-Nefkens et al., 2021). That may have benefited the comprehensibility and the ease with which people filled out the questionnaires.

The completed questionnaires showed a few missing values. Although the analysis did not reveal a pattern, it was notable that most missing values occurred for Item 12 in both the ECD-P and the ECD-C and on both measurements. Researchers’ personal notes showed that for this item (“People in my social environment adjust to my communication problems”) participants made remarks on missing a “not applicable” option. We think this was due to the fact that the participants in this study were people with early-stage dementia and not all of them might have experienced or acknowledged communication problems yet. However, this questionnaire was designed to evaluate an intervention program for people with established communication problems, so we kept this particular item.

We anticipated that the reproducibility of the ECD-P would be moderate, because a condition like dementia can make it more difficult for a person to respond consistently, and also because the second measurement was conducted in a different setting because it was not possible to get all participants to come back to the hospital for a second time within 2 weeks. All parts of the ECD-C have a good test–retest reliability. However, the reproducibility of the ECD-P Part 1 was moderate, while the reproducibility of the second part (two general questions) was even poor. Although the questionnaires for the first and second measurement were equal, we did see some differences on the individual level. This indicates that the way of administering the ECD could have influenced the outcomes either positively or negatively, because people might have discussed the items with each other and influenced each other. This effect might be even greater for people with dementia because they are more likely to ask for assistance from their caregivers and thus potentially be influenced by them. Also, experiences can vary between two measurements, and this difference might be even greater for people with dementia because cognitive limitations and reduced insight interfere with the ability to look back over a longer period of time to give an overall impression of their experiences. This might be especially true for the two abstract single questions like the ones in ECD-P Part 2. Therefore, it was not surprising that this part has a low score in the reliability analysis. Our recommendation for further use is to delete ECD-P Part 2 and to use ECD-P Part 1 always in combination with ECD-C. This decision was also supported by the poor construct validity of the ECD-P2 and its relatively low internal consistency (0.66). Internal consistency was acceptable for all other parts of the questionnaire, justifying the use of sum scores.

The substantial correlation between ECD-P Part 1 and ECD-C Part 1 (*r* = 0.55) indicates that they measure the same construct, but certainly are not interchangeable. Cognitive decline can make it more difficult to answer questions about experienced communication, but our overall results justify the assessment of ECD-P Part 1. Moreover, we think this is worthwhile because the ECD not only aims to measure change due to the intervention, but responses to the items also give direction to the content of the intervention by the speech–language therapist. Other studies underline the feasibility, value, and importance of hearing the voices of persons with dementia, despite that it might demand more preparation, time, and patience from health care professionals or researchers (Perfect et al., 2021; Trigg et al., 2007), and using twin questionnaires is already common practice in dementia care research with quality of life questionnaires (Logsdon et al., 1999; Römhild et al., 2018).

Despite intensive searching, we did not find any self-administered instruments that measure (aspects of) the construct of “experienced communication.” Some recently published proxy-based or observer-rated communication instruments might be suitable for comparison. However, the Threadgold Communication Tool (TCT; Strøm et al., 2016) and the Communication Assessment for Advanced Dementia (CASAD; Volicer & Manzar, 2018) are validated only for people with moderate to severe dementia. Another measure, the Verbal and Nonverbal Interaction Scale (VNVIS-CR; Williams et al., 2017), consists of scoring 13 sociable and 13 unsociable communication behaviors, including verbal and nonverbal items, from 10-min video recordings, which is time-consuming. Apart from the fact that the TCT, CASAD, and VNVIS-CR were not published at the time of data collection for this study, their aforementioned characteristics make them less suitable as convergent measures for the ECD. To appraise construct validity, we chose convergent measures for quality of life and caregiver burden as well as divergent measures for cognitive and physical functioning. As expected, we found substantial correlations in the expected directions between the scores on the ECD and the measures for quality of life of both the person with dementia and the caregiver. Social interaction takes place during a large part of daily life. If social contacts are negatively influenced by communication difficulties (e.g., miscommunication or communication breakdown), this can cause stress, frustration, sadness, and anger, resulting in a lower perceived quality of life (Banerjee et al., 2010; Stiadle et al., 2014). The substantial correlations between the three parts of ECD-C and ZBI-12 indicate that experienced communication and caregiver burden are different, but related concepts. This is in accordance with Savundranayagam et al. (2005), who also found that this relationship is mediated by problem behaviors that can occur as a consequence of communication problems. Interestingly, we expected a weak but significant correlation between the first parts of the ECD and scores on the MMSE, ADL, and IADL, but none of these comparisons showed a significant correlation. This suggests that cognitive and physical functioning are not related to experienced communication. This finding is supported by studies on couple identity and dyadic adaptation to the challenges that arise from one partner having dementia, which indicate that the quality of a relationship is more related to people’s experiences on topics like communication than the actual cognitive impairments (Hernandez et al., 2019; Martin et al., 2009). Obviously, further research is needed in order to fully appraise the construct validity of the ECD.

There are several limitations to consider. First, the questionnaire was designed for people who are referred for logopedic treatment, because they experienced communication disorders as a consequence of dementia. It was, however, difficult to find such a group of people, because referral of persons with dementia to a speech-language therapist is still not very common. Therefore, most people with dementia in this validation study had fairly high scores on the measures for cognitive and physical functioning, and not all of them needed treatment for communication problems at the time of the study. A lack of variety in our population might explain the results of the analyses for construct validity, but we did not find floor effects. This finding is supported by studies showing that communication difficulties can be present from the earliest stages of dementia, even if they are not always acknowledged (de Carvalho & Mansur, 2008). We are convinced that it is beneficial to administer questionnaires like the ECD in the early stages of dementia. Results provide insight for both the person with dementia and the caregiver and can be used to monitor the communication difficulties over time.

Second, we cannot rule out selection bias because we did not collect data on the people who chose not to participate in the study. This might have led to a more homogeneous sample. Lastly, the retest was done at home, without a researcher present. This might have influenced the scores either in a positive or negative direction. To enhance reliability, we recommend administering the ECD in the presence of a researcher or trained health care professional, who can explain the items if necessary or conduct the ECD as an interview, while also minimizing interaction between the person with dementia and the caregiver.

Overall, we conclude that the ECD seems to have promise as a tool to measure experienced communication between persons with early-stage dementia and their caregivers, when ECD-P Part 2 is deleted. Results of this study on clinimetric properties justify future research regarding the use of the ECD in dyads, where communication problems are already established by a health care professional or reported by people themselves. Further investigation in a pre- to postintervention study is needed to determine whether the ECD is able to detect clinically meaningful improvement in experienced communication, also when measuring people in more advanced stages of dementia.

## Supplementary Material

gnab187_suppl_Supplementary_Material

## Data Availability

The data that support the findings of this study are available on request from the corresponding author.
