# Supplementary material for: Clinimetric Evaluation of the Experienced Communication in Dementia Questionnaire
Source: Gerontologist. 2021 Dec 28;63(1):40–51. doi: 10.1093/geront/gnab187 (PMC9872762; doi:10.1093/geront/gnab187)
Supplement: gnab187_suppl_Supplementary_Material [file gnab187_suppl_supplementary_material.docx]

*The Gerontologist* Online Supplementary Material: Olthof-Nefkens, M.W.L.J., Derksen, E.W.C., Lambregts, B., de Swart, B.J.M., Nijhuis-van der Sanden, M.W.G., & Kalf, J.G. Clinimetric Evaluation of the Experienced Communication in Dementia Questionnaire (ECD)

**Section I:**

**English version of the Experienced Communication in Dementia questionnaire – patient version (ECD-P)**

This questionnaire is about how you currently experience the interaction between yourself and the people around you.

Please take the time to read the questions and circle your answer. There are no right or wrong answers!

If you need help, please ask the research assistant or your caregiver for help. Always respond with the answer that best reflects your own opinion.

**Continue on the backside**

© Radboudumc 2021

| To what extent do you agree to the following statements? |  |
| --- | --- |
| 1. My caregiver makes an effort to understand me | strongly disagree - disagree - agree - strongly agree |
| 2.My caregiver usually talks at a pleasant pace  (not too fast and not too slow) | strongly disagree - disagree - agree - strongly agree |
| 3. My caregiver makes eye contact when we talk to each other | strongly disagree - disagree - agree - strongly agree |
| 4. I feel safe in conversations where my caregiver is present | strongly disagree - disagree - agree - strongly agree |
| 5. My caregiver and I talk less and less to each other | strongly disagree - disagree - agree - strongly agree |
| 6. I have become more quiet than I used to be | strongly disagree - disagree - agree - strongly agree |
| 7. I tend to withdraw from conversations | strongly disagree - disagree - agree - strongly agree |
| 8. I try to avoid events where there are many people present | strongly disagree - disagree - agree - strongly agree |
| 9. I like to be helped when I experience communication breakdown | strongly disagree - disagree - agree - strongly agree |
| 10. I tell people when I get stuck in a conversation | strongly disagree - disagree - agree - strongly agree |
| 11. I tell people about my illness | strongly disagree - disagree - agree - strongly agree |
| 12. People adjust to the way I communicate | strongly disagree - disagree - agree - strongly agree |
| 13.I am satisfied with my current social contacts | strongly disagree - disagree - agree - strongly agree |
| 14. Friends and acquaintances come to visit as often as they did in the past | strongly disagree - disagree - agree - strongly agree |

**Turn page**

© Radboudumc 2021

| How often do the following situations occur: |  |
| --- | --- |
| 15. I can’t find the right words | during every conversation - every day - every week - (almost) never |
| 16. I am not able to participate because the conversation goes too fast | during every conversation - every day - every week - (almost) never |
| 17. There are misunderstandings between me and my caregiver | during every conversation - every day - every week - (almost) never |
| 18. I feel nervous during a conversation | during every conversation - every day - every week - (almost) never |
| 19.I feel frustrated during a conversation | during every conversation - every day - every week - (almost) never |
| 20. I feel sad during a conversation | during every conversation - every day - every week - (almost) never |
| 21. I feel angry during a conversation | during every conversation - every day - every week - (almost) never |
| 22. I feel anxious during a conversation | during every conversation - every day - every week - (almost) never |

**Thank you very much for filling out this questionnaire!**

© Radboudumc 2021

**Section II:**

**English version of the Experienced Communication in Dementia questionnaire – caregiver version (ECD-C)**

This questionnaire is about how you currently experience the interaction between your partner (or read here: father/mother/the person for whom you are a caregiver) and the people in his or her social environment.

Please take the time to read the questions and answer them. There are no right or wrong answers!

If you need help, please ask the research assistant for help. Always respond with the answer that best reflects your own opinion.

**Continue on the backside**

© Radboudumc 2021

| To what extent do you agree to the following statements? |  |
| --- | --- |
| 1. I make an effort to understand my partner | strongly disagree - disagree - agree - strongly agree |
| 2.I usually talk at a pleasant pace (not too fast and not too slow) | strongly disagree - disagree - agree - strongly agree |
| 3. I make eye contact with my partner when we talk to each other | strongly disagree - disagree - agree - strongly agree |
| 4. My partner feels safe in conversations where I am present | strongly disagree - disagree - agree - strongly agree |
| 5. My partner and I talk less and less to each other | strongly disagree - disagree - agree - strongly agree |
| 6. My partner has become more quiet than he/she used to be | strongly disagree - disagree - agree - strongly agree |
| 7. My partner tends to withdraw from conversations | strongly disagree - disagree - agree - strongly agree |
| 8. My partner tries to avoid events where there are many people present | strongly disagree - disagree - agree - strongly agree |
| 9. My partner likes to be helped when he/she experiences communication breakdown | strongly disagree - disagree - agree - strongly agree |
| 10. My partner tells people when he/she gets stuck in a conversation | strongly disagree - disagree - agree - strongly agree |
| 11. My partner tells people about his/her illness | strongly disagree - disagree - agree - strongly agree |
| 12. People adjust to the way my partner communicates | strongly disagree - disagree - agree - strongly agree |
| 13.My partner is satisfied with his/her current social contacts | strongly disagree - disagree - agree - strongly agree |
| 14.Friends and acquaintances come to visit as often as they did in the  past | strongly disagree - disagree - agree - strongly agree |

**Turn page**

© Radboudumc 2021

| How often do the following situations occur: |  |
| --- | --- |
| 15. My partner can’t find the right words | during every conversation - every day - every week - (almost) never |
| 16.My partner is not able to participate because the discussion goes too fast. | during every conversation - every day - every week - (almost) never |
| 17. There are misunderstandings between me and my partner | during every conversation - every day - every week - (almost) never |
| 18. My partner feels nervous during a conversation | during every conversation - every day - every week - (almost) never |
| 19. My partner feels frustrated during a conversation | during every conversation - every day - every week - (almost) never |
| 20. My partner feels sad during a conversation | during every conversation - every day - every week - (almost) never |
| 21. My partner feels angry during a conversation | during every conversation - every day - every week - (almost) never |
| 22. My partner feels anxious during a conversation | during every conversation - every day - every week - (almost) never |
|  |  |
| 23.In general, I would grade the conversations between me and my partner with an: | (poor) 1 – 2 – 3 – 4 – 5 – 6 – 7 – 8 – 9 – 10 (excellent) |
| 24.In general, I would grade the conversations between my partner and the people in our immediate surroundings (children, friends, neighbours, etc.) with an: | (poor) 1 – 2 – 3 – 4 – 5 – 6 – 7 – 8 – 9 – 10 (excellent) |

**Continue on the backside**

© Radboudumc 2021

| Respond to the following statements: |  |
| --- | --- |
| 25. I find it tiring to interact with my partner | strongly disagree - disagree - agree - strongly agree |
| 26. It burdens me that communication is becoming increasingly difficult | strongly disagree - disagree - agree - strongly agree |
|  |  |
| 27. I feel angry during a conversation | during every conversation - every day - every week - (almost) never |
| 28. I feel sad during a conversation | during every conversation - every day - every week - (almost) never |
| 29. I feel frustrated during a conversation | during every conversation - every day - every week - (almost) never |

**Thank you very much for filling out this questionnaire!**

© Radboudumc 2021
